# Medical Literature of Kentucky

**Published:** 1853-04

**Authors:** 


### MEDICAL LITERATURE OF KENTUCKY.

At the last meeting of the Medical Society of Kentucky the senior editor of this *Journal* was appointed Chairman of the Committee on the *Medical Literature*, or *the History of the Medical Authorship of Kentucky;* and has to request those who have any knowledge of the early medical history of the State, to favor him with communications on the subject. Books, pamphlets, documents of whatever character, written by Kentucky physicians, or any information concerning any that may be out of print and inaccessible, would be particularly acceptable. The wish of the Chairman of the Committee is, to give a full history of the medical authorship of the State.

